# 12-month follow-up of an exploratory ‘brief intervention’ for high-frequency cannabis users among Canadian university students

**DOI:** 10.1186/1747-597X-7-15

**Published:** 2012-04-26

**Authors:** Benedikt Fischer, Wayne Jones, Paul Shuper, Jürgen Rehm

**Affiliations:** 1 Centre for Applied Research in Mental Health and Addictions, Faculty of Health Sciences, Simon Fraser University, 2400, 515 West Hastings St., Vancouver, BC, V6B 5K3, Canada; 2 Centre for Addiction and Mental Health, 33 Russell St., Toronto, ON, M5S 2S1, Canada; 3 Dalla Lana School of Public Health, University of Toronto, 155 College St., Toronto, ON, M5T 3M7, Canada

**Keywords:** Cannabis use, Frequent use, Young adults, Brief interventions, Prevention, Canada

## Abstract

**Background:**

One in three young people use cannabis in Canada. Cannabis use can be associated with a variety of health problems which occur primarily among intensive/frequent users. Availability and effectiveness of conventional treatment for cannabis use is limited. While Brief Interventions (BIs) have been shown to result in short-term reductions of cannabis use risks or problems, few studies have assessed their longer-term effects. The present study examined 12-month follow-up outcomes for BIs in a cohort of young Canadian high-frequency cannabis users where select short-term effects (3 months) had previously been assessed and demonstrated.

**Findings:**

N = 134 frequent cannabis users were recruited from among university students in Toronto, randomized to either an oral or a written cannabis BI, or corresponding health controls, and assessed in-person at baseline, 3-months, and 12-months. N = 72 (54 %) of the original sample were retained for follow-up analyses at 12-months where reductions in ‘deep inhalation/breathholding’ (Q = 13.1; p < .05) and ‘driving after cannabis use’ (Q = 9.3; p < .05) were observed in the experimental groups. Reductions for these indicators had been shown at 3-months in the experimental groups; these reductions were maintained over the year. Other indicators assessed remained overall stable in both experimental and control groups.

**Conclusions:**

The results confirm findings from select other studies indicating the potential for longer-term and sustained risk reduction effects of BIs for cannabis use. While further research is needed on the long-term effects of BIs, these may be a valuable – and efficient – intervention tool in a public health approach to high-risk cannabis use.

## Background

Cannabis use is prevalent in Canada; about one in ten adults are current users [1,2]. Use is disproportionately prevalent among young people (e.g., 15 – 24 years of age) including secondary and post-secondary students, where some 25 % - 36 % are current cannabis users [1,3]. A variety of health risks – including cognitive and psychomotor impairment, bronchial or pulmonary problems, mental health and dependence, injuries - are associated with cannabis use [4,5]. However, these problems disproportionately occur in a minority of users [6,7]. More specifically, intensive (e.g., frequent/daily) users are at high risk for above problems and hence form a primary target group for interventions [6,8,9].

Traditional treatment interventions are limited in availability and effectiveness. In this context, the utility of so-called ‘Brief Interventions’ (BIs) for reducing risk or problem outcomes from cannabis use has recently been examined. BIs are largely modelled after similar efforts in other substance use areas, mainly alcohol, where reductions in risk behaviors have been shown [10-12]. BIs for cannabis use usually consist of a small number of intervention units, delivered in varying formats, conveying information and/or motivational components towards reducing risk behaviors or problems [13,14]. BIs for cannabis use have been shown to achieve substantive short-term reductions in, for example, cannabis use frequency or problem indicators (e.g., dependence symptoms); however information on longer-term effects is limited [15,16].

The objective of this present study was to assess the longer-term (i.e., 12-months) impact of a customized BI module for cannabis use on select risk outcome indicators, for which short-term (i.e., 3-months) impact was previously assessed and demonstrated, in frequent cannabis users among university students in a Canadian setting.

## Methods

### Sample and recruitment

Details of the study methods are provided in [17]. Briefly, participants were recruited from university student populations in Toronto by way of mass-advertising (i.e., posters on campuses) based on the following eligibility criteria: 18 – 28 years of age; full-time university enrolment; cannabis use > one year; cannabis use on at least 12 of the past 30 days. Study eligibility confirmation occurred by telephone, after which candidates were invited for the in-person and anonymous study appointment.

### Assessment and interventions

Baseline assessments consisted of a short (25–30 minutes) interviewer-administered questionnaire after which participants were randomized to one of four BI groups: a cannabis (experimental) intervention delivered either orally (C-O) or in the form of written material (C-W), or a general health (control) intervention delivered orally (H-O) or via written material (H-W). The cannabis interventions consisted of short information elements on cannabis-related health risks, concrete suggestions for risk modification, and brief motivational components; the main aims of the BI were to inform users about key modifiable health risks related to use, and to suggest tangible ways to reduce these risks without principally focusing on or requiring abstinence. The control interventions were similarly structured and included general health information content (e.g., nutrition, stress, exercise). The oral BIs were delivered by a health psychologist in a 15 – 20 minutes long session; the written BIs were provided by a 10-page booklet with corresponding content. Follow-up assessments were conducted in-person at 3-months (FU3) and 12-months (FU12) following the BI, consisting of an abbreviated version of the baseline interview.

Participants provided informed consent for all study elements, and received small honoraria for baseline ($25) and follow-up assessments ($30 each). The study was approved by the investigators’ institutional research ethics board.

### Measures and analyses

Four measures (all ‘in the past 30 days’) collected at baseline and follow-up assessments were used to assess changes in key outcomes:

(1) Number of cannabis use days (continuous).

(2) Average number of cannabis use episodes per use day (continuous).

(3) Use of deep inhalation/breathholding techniques (yes/no).

(4) Driving a vehicle within 2 hours of use (yes/no).

The FU3 analysis found little difference between the oral and written BI modules, and thus the respective experimental and control groups were collapsed (i.e., C-O + C-W and H-O + H-W) for analyses. On this basis, the two continuous measures were analysed by a 2 (intervention group [cannabis or health]) by 3 (measurement period [baseline, FU3, FU12]) ANOVA with repeated measures over period. To account for the correlated nature of the repeated measures the two categorical measures were examined using Cochran’s Q test (for changes over the 3 periods) and the more detailed comparisons between baseline and FU3, and FU3 and FU12 by McNemar’s chi-square.

## Findings

### Study sample

**Table 1:** Key socio-demographic and cannabis use characteristics of baseline, completer and lost-to-follow-up samples, by intervention group

**Sample**	**Intervention Group**		**Age**	**Years of University**	**Years of Cannabis Use**	**Sex**
		N	Mean (sd)	Mean (sd)	Mean (sd)	%Male
*Baseline Sample*	All	134	20.4 (2.4)	2.5 (1.6)	5.5 (2.7)	67.2 %
	Cannabis BI	72	20.3 (2.4)	2.4 (1.7)	5.5 (2.8)	65.3 %
	Health BI	62	20.6 (2.5)	2.6 (1.5)	5.6 (2.6)	69.4 %
*Completer (baseline, FU-3 and FU-12) Sample*	All	72	20.3 (2.2)	2.5 (1.4)	5.7 (2.8)	68.1 %
	Cannabis BI	40	20.1 (2.1)	2.3 (1.5)	5.9 (3.0)	67.5 %
	Health BI	32	20.6 (2.3)	2.7 (1.3)	5.5 (2.6)	68.8 %
*Lost-to-Follow-up Sample*	All	62	20.6 (2.7)	2.5 (1.7)	5.3 (2.6)	66.1 %
	Cannabis BI	32	20.5 (2.7)	2.6 (1.8)	5.0 (2.4)	62.5 %
	Health BI	30	20.6 (2.7)	2.5 (1.6)	5.7 (2.7)	70.0 %

The baseline sample consisted of n = 134 subjects. Of these, n = 113 (84 %) were reassessed at FU3, and n = 76 (57 %) were reassessed at FU12. Four subjects of the FU12 sample had not attended the FU3 assessment, meaning complete data for all three assessments resulted in an analysis (completer) sample of n = 72 (54 %). Table 1 presents data on key socio-demographic characteristics (e.g., age, sex, years of university enrolment) and years of cannabis use for the baseline and completer samples and those lost to follow up (LTF). No differences were found for the above indicators between the completer and the LTF samples.

### Outcomes

**Table 2:** Key study outcomes among completer sample, by intervention group, at baseline, 3-month follow-up (FU3) and 12-month follow-up (FU12)

**Measure**	**Group**	**N**	**Mean (95 % CI)**		
			**Baseline**	**FU 3**	**FU 12**
*Number of days cannabis used*	Cannabis	40	24.0 (22.2-25.8)	23.1 (20.7-25.5)	22.3 (19.8-24.8)
	Health	32	23.9 (21.8-26.0)	23.1 (20.6-25.5)	22.1 (18.9-25.3)
*Number of cannabis use episodes/day*	Cannabis	40	2.3 (1.7-2.9)	2.4 (1.8-3.0)	2.6 (1.6-3.7)
	Health	32	2.0 (1.7-2.3)	2.4 (1.5-3.4)	2.2 (1.7-2.6)
			**Proportion Responding YES**		
		**N**	**Baseline**	**FU 3**	**FU 12**
*Deep inhalation/ breathholding*	Cannabis*	40	78 %	52 %	57 %
	Health	32	81 %	78 %	68 %
*Driving after cannabis use*	Cannabis*	40	44 %	31 %	24 %
	Health	32	30 %	27 %	24 %

* Significant change over periods (p < .05).

The repeated measures ANOVAs found no statistically significant main or interaction effects for ‘cannabis use days’ or the ‘cannabis use episodes’. A change was observed for ‘deep inhalation/breathholding’ over the year for the experimental (Q = 13.1; p < .05) but not the control (Q = 4.8; ns) group. Similarly, a change was observed for ‘driving after cannabis use’ for the experimental (Q = 9.3; p < .05) but not the control (Q = 0.9; ns) group. Changes observed occurred in the desired direction, i.e., reductions in risk behaviours. The most substantive changes for these variables had materialized between baseline and FU3; these effects were maintained at the FU12 assessments. Also, for the ‘driving after cannabis use’ variable, randomization appears to have resulted in a higher proportion in the experimental group at baseline reporting “Yes”; however this dropped to the level of the control group by FU3 and this effect was maintained over the year (see Table 2).

No significant differences were detected for the above outcome variables at baseline between the experimental and control samples or between the completer and LTF samples.

## Discussion

Our results suggest that reductions in two of four key risk outcome indicators following a BI delivered to high-frequency young cannabis users observed at FU3 (i.e., deep inhalation/breathholding and driving after cannabis use) were maintained at FU12. These results are, in principle, consistent with findings from the (few) other studies on longer term effects of BIs, suggesting that reductions in specific risk behaviors from cannabis BIs may be sustained beyond short-term periods. For example, Dennis et al. [18] found substantial increases in the number of abstinence days following several BIs for adolescent cannabis use maintained after one year; Stephens et al. [16] and Babor et al. [19] found post-BI reductions in cannabis use days and problem symptoms maintained for at least one year. Notably, our study did not see changes in cannabis use frequency or intensity itself, as witnessed by the above studies, but reductions were limited to the (rather specific) risk behavior variables. It needs further investigation to which extent this may be a function of the specific BI content applied, or possibly related to participants’ dispositions that frequent cannabis use itself is not a behavior worthy of change.

While relevant impacts from BIs appear to materialize immediately following the interventions, the present longer-term outcomes may be viewed as an indication of the stability of such effects. On this basis, our results add to the growing evidence that BIs may be a potentially effective – and efficient - tool in decreasing key cannabis related risk behaviours relevant for individual and public health [10,12,20]. Specifically, deep inhalation/breathholding may increase the risk for pulmonary/bronchial problems, and driving after cannabis use is likely to result in impaired driving, associated with elevated risk for motor vehicle accident involvement and/or injury [4,9,21,22]. While the demonstrated reductions are limited to select indicators, and do not eliminate the specified risk behaviors, conventional - and more resource-intensive - treatment interventions do not appear to result in substantially more efficacious impacts [10,12].

The sustained impact and intervention utility of BIs beyond just ‘strawfire’ effects may further be amplified – or extended to other behaviors - by way of implementing targeted ‘booster’ interventions, e.g., by way of follow-up calls, text messages, etc. [18,23]. Given their relative ease and flexibility in delivery, BIs can relatively easily be offered to high-risk groups in their natural environments or settings (e.g., universities) [20,24,25]

The results of the present study should be further tested in larger-scale studies examining different BI content and delivery method options with sufficient power and longer-term follow-ups, as well as ideally relying on an intent-to-treat (rather than a completers_) analysis approach. Key limitations of the present study include its small sample size, reducing the sensitivity of our methods to detect potential effects. Specifically, the health controls seemed to indicate trends towards reductions in the risk factors under discussion that may have evolved to significance given more analytical power. In addition, there may have been un-measured extrinsic factors over the study period influencing participants’ risk behaviors that could not be accounted for in the analysis. The study had a relatively substantial loss to follow up – largely due to high mobility in student cohorts – which could lead to selection biases, although we found no relevant differences between the analysis and LTF samples. The study relied primarily on self-reported data, the validity of which can be confounded (e.g., social desirability effects). The results of the study may not be generalizable to other cannabis user populations.

Overall, our study suggests the potential utility of BIs as an intervention tool in Canada where cannabis use is prevalent among young people, and BIs have largely been ignored in the substance use arena to date. In the context of increasing advocacy for public health oriented cannabis use policy in Canada, BIs may further be developed and utilized as a valuable intervention tool, primarily for secondary prevention purposes, for key risk populations [4,20]. At this point, the vast majority of intervention resources for cannabis use control in Canada are expended for criminalization (e.g., arrests of users); in addition, large-scale abstinence education or promotion campaigns are implemented with questionable impact. A small proportion of the resources used for the aforementioned programs would allow broad-based BI programs to be implemented for high-risk cannabis users in settings (e.g., universities, colleges) where such populations are disproportionately present and offer themselves well for such measures, as has been shown for other public health oriented intervention campaigns (e.g., sexual health education or nutritional education, etc.). In addition, other key stakeholders – e.g., non-governmental public health associations – could become involved in offering such BI programming.

## Competing interests

The authors declare that they have no competing interests.

## Authors’ contribution

BF overall led the study. BF, JR and PS designed the study protocol. WJ, BF, PS and JR designed the analysis plan; WJ conducted the data analysis. All authors contributed to interpretation of the data, and to drafting and revising the present manuscript.

## Role of funding sources

The authors acknowledge funding support from the Canadian Institutes of Health Research (CIHR), specifically Catalyst Grant #211803, for this study. Drs. Fischer, Rehm and Shuper also acknowledge funding support from the Ontario Ministry of Health and Long-Term Care (MOHLTC). Dr. Fischer furthermore acknowledges salary support from a CIHR/PHAC Chair in Applied Public Health.
